# Effect of d-limonene and its derivatives on breast cancer in human trials: a scoping review and narrative synthesis

**DOI:** 10.1186/s12885-021-08639-1

**Published:** 2021-08-06

**Authors:** Joy J. Chebet, John E. Ehiri, Deborah Jean McClelland, Douglas Taren, Iman A. Hakim

**Affiliations:** 1 Department of Health Promotion Sciences, Mel and Enid Zuckerman College of Public Health, University of Arizona, 1295 N Martin Ave, Tucson, AZ 85724 USA; 2 University of Arizona Health Sciences Library, University of Arizona, Tucson, AZ USA

**Keywords:** Citrus peel, D-limonene, Perillyl alcohol, Breast cancer, Chemopreventive, Scoping review

## Abstract

**Background:**

D-limonene and its derivatives have demonstrated potential chemopreventive and anticancer activity in preclinical and clinical studies. The aim of this scoping review was to assess and critically appraise current literature on the effect of these bioactive citrus peel compounds on breast cancer in human trials and to identify knowledge gaps for exploration in future studies.

**Methods:**

This study followed a scoping review framework. Peer-reviewed journal articles were included if they reported the effect of d-limonene or its derivatives on breast cancer in human subjects. Articles were retrieved from academic databases – PubMed, EMBASE, CINAHL, Web of Science, and Cochrane reviews – and iteratively through review of bibliographies of relevant manuscripts. Titles and abstracts were appraised against the aforementioned inclusion criteria in a first round of screening. Through consensus meetings and full article review by authors, a final set of studies were selected. Results were reported according to the PRISMA extension for scoping reviews.

**Results:**

Our search strategy yielded 367 records. Following screening and adjudication, five articles reporting on phase 1(*n* = 2), phase 2 (n = 2) and both trial phases (*n* = 1) were included as the final dataset for this review. Trials evaluating the effect of d-limonene (*n* = 2) showed it was well tolerated in subjects. One study (*n* = 43 participants) showed d-limonene concentrated in breast tissue (mean 41.3 μg/g tissue) and reduction in tumor cyclin D1 expression, which is associated with tumor proliferation arrest. This study did not show meaningful change in serum biomarkers associated with breast cancer, except for a statistically significant increase in insulin-like growth factor-1 (IGF-I) levels. While elevation of IGF-I is associated with increased cancer risk, the clinical implication of this study remains uncertain given its short duration. Trials with perillyl alcohol (*n* = 3) showed low tolerance and no effect on breast cancer.

**Conclusion:**

This review demonstrated a dearth of clinical studies exploring the effect of d-limonene and its derivatives on breast cancer. Limited literature suggests d-limonene is safe and tolerable in human subjects compared to its derivative, perillyl alcohol. Our review demonstrates the need for additional well-powered placebo-controlled trials that assess d-limonene’s efficacy on breast cancer compared to other therapies.

**Supplementary Information:**

The online version contains supplementary material available at 10.1186/s12885-021-08639-1.

## Background

Breast cancer is the second most common cancer among women in the United States, accounting for approximately 30% of all cancers diagnosed in this population [1]. In 2021, an estimated 284,200 new cases and 44,130 deaths attributable to breast cancer are projected [1]. Depending on a confluence of factors – ranging from age, cancer type, and cancer stage – clinical treatment options for breast cancer include surgical interventions, chemotherapy, hormonal, biological, and radiation therapies. These currently available therapies are invasive, associated with severe side effects, and/or are expensive to administer [2–4]. There is therefore a need for cheaper and more tolerable alternative treatments.

Citrus fruits, including lemons, limes, oranges, tangerines, and grapefruits, are widely available at low cost. These fruits contain bioactive compounds – including phenols, flavonoids and terpenes – which have demonstrated chemotherapeutic properties in relation to breast cancer [5–8]. While other compounds in the citrus peel have been evaluated for anticancer activity, limonene, the simplest monocyclic monoterpene, shows substantial chemotherapeutic promise because: (1) it constitutes 3.8 wt% of dry orange peel, and about 90–95% of citrus oil [9]; (2) it is fat-soluble, allowing for absorption into fat tissue, thereby permitting for the monoterpene to accumulate in the body [10]; and (3) it is rapidly metabolized in humans in a fashion similar to animal models [11].

**Fig. 1:**
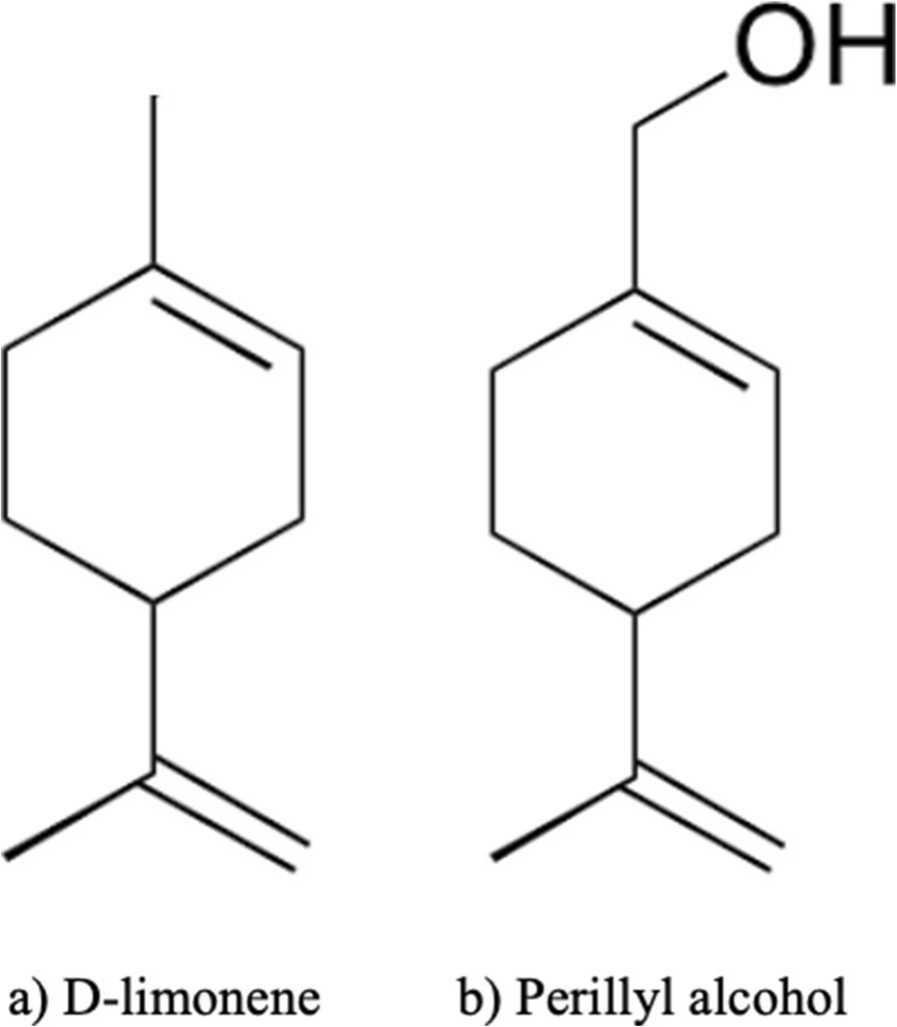
Chemical structure of d-limonene and perillyl alcohol

Limonene (Fig. 1) has demonstrated clinical and therapeutic applications [12, 13]. Among humans, limonene has been shown to be effective in dissolving gallstones [14], and in relieving Gastroesophageal Reflux Disease (GERD) symptoms [12]. Exploration into hydroxylated analogs of d-limonene – including perillyl alcohol, carveol, uroterpenol, and sobrerol – have been investigated, owing to their efficacy in causing tumor regression in animal models at lower concentrations than d-limonene [15, 16]. Their use, therefore, have been postulated as resulting in more favorable therapeutic ratios [15]. Perillyl alcohol (Fig. 1), a dietary monoterpene is found naturally in peppermint, lavender, and other plants.

Given the positive results of in vivo and in vitro studies, chemotherapeutic evaluation of d-limonene and perillyl alcohol have progressed to human studies. To our knowledge, findings from these clinical studies have not been consolidated into a single review, which would allow for a comprehensive mapping of literature on the subject, as well as a repository of this evidence. The objectives of this review, therefore, are to explore the depth of knowledge currently available on the effect of d-limonene and its derivatives on breast cancer in humans. Specifically, this scoping review aims to: (1) systematically review and summarize evidence on citrus peel extracts and breast cancer in humans, including populations that have been studied, tolerable dosage, observed toxicity, pharmacokinetic profiles and anticancer effects; and (2) discuss studied underlying mechanisms leading to anticancer activity.

## Methods

### Inclusion criteria and search strategy

The design of this review was informed by Arksey and O’Malley’s methodological framework for scoping reviews [17]. Peer-reviewed journal articles were included in the present study if they: (1) reported on the effect of d-limonene or any of its derivatives; (2) measured breast cancer as a key outcome; (3) were conducted among human subjects; 4) employed an experimental design, with or without a comparison; and (5) were published at any time before June 20, 2020. Studies were excluded if they: (1) were not peer-reviewed; and (2) did not present original data.

A medical librarian collaborated on this review and contributed to the development of the search strategy. Academic databases – Legacy PubMed, Embase, EBSCOhost Cumulative Index of Nursing and Allied Health Literature (CINAHL), Web of Science (Core Databases), and Cochrane Reviews – were searched to retrieve academic peer-reviewed journal articles. To capture articles relevant to the review questions, a search strategy incorporating key words and controlled vocabulary pertinent to our exposure (d-limonene OR citrus oil OR orange oil OR Lemon oil OR Mandarin oil OR Lime oil OR Grapefruit oil OR citrus peel OR carveol OR uroterpenol OR sobrerol OR “limonene”[MeSH] OR “citrus”[MeSH] OR “citrus paradise”[MeSH]) and outcome (breast cancer OR breast carcinoma OR mammary cancer OR cancer of the breast OR “breast neoplasms”[MeSH]) was used (supplemental Table 1). In an iterative process, involving the review of bibliographies of relevant manuscripts, additional articles were included to ensure an exhaustive search. Articles retrieved from the academic databases and bibliography review were combined, and duplicates removed to arrive at a consolidated dataset to determine eligibility for inclusion in the review.

### Data management and extraction

All article citations were managed using the Mendeley reference manager. To allow for collaboration and transparency through the screening process, Rayyan QCRI, a web- and mobile-based systematic review application was used [18]. In an initial round of screening, study authors reviewed the titles and abstracts in the consolidated dataset for relevance based on the abovementioned inclusion/exclusion criteria. Following this first review, authors convened to discuss the articles resulting from the first screening and came to a consensus about the articles to be excluded. In a secondary screening, articles were reviewed in their entirety and included in the present review if they met the eligibility criteria. Queries on the eligibility for inclusion were resolved through consensus of authors. A final set of articles fitting the scope of the present review were analyzed and summarized.

### Data analysis and synthesis

To assess methodological quality, the Joanna Briggs Institute (JBI) Checklist for Quasi-Experimental Studies (non-randomized experimental studies) was applied to the final set of articles (supplemental Table 2) [19]. Additionally, the quality of each included article was assessed using the Risk Of Bias In Non-randomized Studies of Interventions (ROBINS-I) Tool [20]. Risk of bias was assessed under the following 7 domains of bias: confounding, selection of participants, classification of interventions, deviation from protocol, missing data, measurement of outcomes and selection of the reported result [20]. For each domain, articles were assigned a gradation of risk of bias – from no information, low, moderate, serious and critical risk.

A summary of each article – consisting of the author and publication details, compound under investigation, research aim and major findings – were performed. Findings were synthesized and categorized based on (1) study participant characteristics; (2) tolerance to monoterpene under evaluation; (3) toxicity to monoterpene under evaluation and corresponding dosing; and (4) effect of monoterpene on breast cancer. Data was reported using PRISMA extension for scoping reviews (PRISMA-ScR) (supplemental Table 3) [21].

## Results

### Included studies

**Fig. 2:**
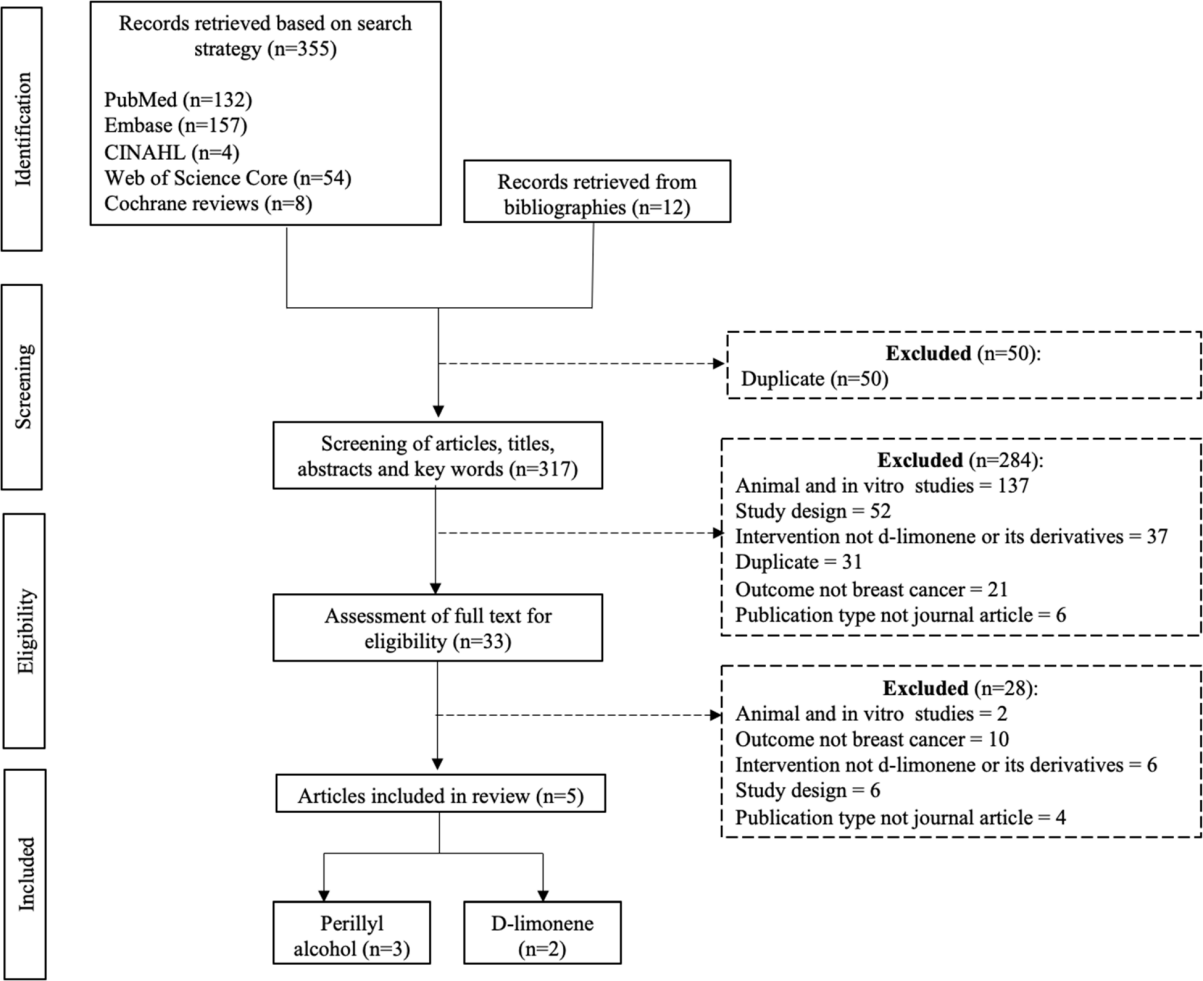
Flow chart illustrating the process of screening and identifying articles included in present review

An initial search yielded 367 records from academic databases (*n* = 355) and review of bibliographies (*n* = 12). Following deduplication, initial screening of titles and abstracts, full text review and researcher adjudication, five articles met the eligibility criteria for inclusion in this present review (Fig. 2). These studies, conducted between 1998 and 2013, included clinical evaluation of d-limonene (*n* = 2) and perillyl alcohol (*n* = 3) on breast cancer-specific outcomes. Included studies reported findings from phase 1 safety and dose escalation (*n* = 3) and phase 2 efficacy (*n* = 3) clinical studies (note: one article included results from both a phase 1 and 2 study). As such, these clinical trials were relatively small (10–43 participants), non-randomized, and did not include a comparison group. Four of the five articles were found to have low overall risk of bias, with one found to be at serious risk of bias due to deviation from the study protocol (Table 1).

**Table 1:** Evaluation of bias in articles included in present review using the Risk Of Bias In Non-randomized Studies of Interventions (ROBINS-I) tool

Bias domain	Vigushin et al. 1998 [22]	Ripple et al. 1998 [23]	Ripple et al. 2000 [24]	Bailey et al. 2008 [25]	Miller et al. 2013 [26]
**Pre-intervention**					
Confounding	low	low	low	low	low
Selection of participants	low	low	low	low	low
**At intervention**					
Classification of intervention	low	low	low	low	low
**Post intervention**					
Deviation from intended intervention	low	low	low	Serious	low
Missing data	low	low	low	low	low
Measurement of outcomes	low	low	low	low	low
Selection of reported results	low	low	low	low	low
**Overall**	low	low	low	Serious	low

### Participant characteristics

One hundred and thirty-three participants were included across the two d-limonene (*n* = 85 participants) and three perillyl alcohol (*n* = 48 participants) studies meeting eligibility for this review (Table 2). However, while 136 total participants were enrolled in the studies, 128 (94%) were evaluable for study outcomes. The 8 participants not evaluated in the studies were removed owing to death, severe adverse effects, non-compliance and disease progression. Three of the studies focused solely on the effect of the monoterpenes d-limonene and perillyl alcohol on breast cancer [22, 25, 26]. The remaining 2 studies included participants with other cancers, in addition to breast cancer [23, 24]. Majority of the participants included in this review were women (73%) and ranged between the ages of 23 and 90. Overall, 83% of the total number of participants in the studies included in this review were diagnosed with breast cancer.

**Table 2:** Summary of collated participant characteristics of participants included in present review

	Vigushin et al. 1998 [22]		Ripple et al. 1998 [23] (*n* = 18)	Ripple et al. 2000 [24] (*n* = 16)	Bailey et al. 2008 [25] (*n* = 14)	Miller et al. 2013 [26] (*n* = 43)	Total (***N*** = 133)
	Phase 1 (*n* = 32)	Phase 2 (*n* = 10)					
Enrolled participants; n	32	10	18	19	14	43	136
Evaluable participants n (%)	32 (100)	10 (100)	16 (88.9)	16 (84.2)	14 (100)	40 (93.0)	128 (94.1)
**Age;** years							
Median	57	57	58.6^a^	50	58	58.5	
Range	35–78	40–82	23–82	24–79	40–90	–	23–90
**Sex**; n (%)							
Male	15 (46.9)	0 (0)	8 (44.4)	13 (81.3)	0 (0)	0 (0)	36 (27.1)
Female	17 (53.1)	10 (100)	10 (55.6)	6 (37.5)	14 (100)	40 (100)	97 (72.9)
**Breast cancer**; n (%)	16 (50.0)	10 (100)	2 (11.1)	1(6.3)	14 (100)	40 (100)	83 (62.4)
**Prior therapies**							
Surgery	29 (90.6)	9 (90.0)	0 (0)	0 (0)	0 (0)	0 (0)	38 (28.6)
Chemotherapy	28 (87.5)	7 (70.0)	14 (77.8)	14 (87.5)	11 (78.6)	0 (0)	74 (55.6)
Radiation therapy	23 (71.9)	7 (70.0)	9 (50.0)	9 (56.3)	0 (0)	0 (0)	48 (36.1)
Hormone therapy	20 (62.5)	10 (100)	6 (33.3)	4 (25.0)	13 (92.9)	6 (14.0)	59 (44.4)
Supportive therapy	2 (6.3)	0 (0)	0 (0)	0 (0)	0 (0)	0 (0)	2 (1.5)
Biologic response modifiers	0 (0)	0 (0)	4 (22.2)	1 (6.3)	0 (0)	0 (0)	5 (3.8)
Endocrine agents	0 (0)	0 (0)	0 (0)	0 (0)	7 (50.0)	0 (0)	7 (5.3)

^a^Only mean age provided in article

- not provided

Majority of the included studies enrolled participants with advanced or metastatic disease [22–25], that were unresponsive to conventional therapies [22–24]. Only one study by Miller et al. enrolled participants with early-stage malignancies [26]. Exclusion criteria included pregnancy/breastfeeding [22, 25, 26], brain metastasis [23, 25], HIV diagnosis [22], and receipt of treatment (hormonal, immunological, chemo or radiation therapies) in weeks preceding participation in study [24–26]. All studies excluded participants using cholesterol lowering drugs, vitamin supplements, and participants with poor kidney or bone marrow function. About half of the participants included in this review (56%) had undergone one or more cycles of chemotherapy prior to enrollment in respective studies. Furthermore, participants had undergone hormone therapy (44%), radiation therapy (36%) and surgery (29%).

### Intervention and supplement formulation

In all studies included in this review, either d-limonene or perillyl alcohol was administered orally as an intervention (Table 3). In phase 1 dose escalation studies, perillyl alcohol was formulated as soft gelatin capsules consisting 250 mg perillyl alcohol and 250 mg soybean oil [23–25]. Dosing was escalated from 800 (level 1), to 1200 (level 2) and 1600 (level 3) mg/m^2^/dose administered 3 times a day in the Ripple et al. (1998) study [23], and 800 (level 1), to 1600 (level 2) and 2400 (level 3) mg/m^2^/dose administered 4 times a day in the Ripple et al. (2000) study [24]. In a more recent phase 2 trial by Bailey et al. [25], 1200 (level 1) and 1500 (level 2) mg/m^2^/dose were administered 4 times a day. Only 1 d-limonene trial by Vigushin et al. [22] included a dose escalation study, where the schedule ranged from 0.5–12 g/m^2^/day. In the second limonene study by Miller et al. [26], 2 g of commercially available d-limonene was administered.

**Table 3:** Summary of clinical studies included in review of d-limonene and its derivatives on breast cancer

Author(s)	Trial Phase	Compound	Cancer type	Dose	Toxicity	Maximum tolerated dose	Effect on breast cancer
**d-limonene**							
Vigushin et al. 1998 [22]	Phase 1: Human subjects (*n* = 32)	Orally administered d-limonene	Phase 1: refractory solid tumors^a^	0.5 to 12 g/m^2^ per day in 21-day cycles	Gastrointestinal toxicities leading to nausea, vomiting and diarrhea were dose limiting	8 g/m^2^ per day	Phase 1: Partial response^b^ observed in one breast cancer patient. Effect was sustained for 11 months.
	Phase 2: Human subjects (*n* = 10)		Phase 2: locally advanced breast cancer	8 g/m^2^ per day for 15 cycles		Not indicated	Phase 2: no response
Miller et al. 2013 [26]	Phase 2: Human subjects (*n* = 43)	Orally administered d-limonene	Newly diagnosed, operable cancers breast cancer	2 g d-limonene for 2–6 weeks	Well tolerated	Not indicated	D-limonene concentrated in breast tissue (mean 41.3 μg/g tissue); Small but statistically significant increase in insulin-like growth factor levels; Reduction in tumor cyclin D1 expression
**Perillyl alcohol**							
Ripple et al. 1998 [23]	Phase 1: Human subjects (*n* = 18)	Orally administered perillyl Alcohol	Advanced malignancies^c^	Dose escalation: 800, 1200 and 1600 mg/m^2^/dose administered 3 time a day	Dose- related gastrointestinal toxicities leading to nausea and vomiting; 2 participants experienced severe drug related myelosuppression	Not indicated	No objective tumor response observed in any patients
Ripple et al. 2000 [24]	Phase 1: Human subjects (*n* = 16)	Orally administered perillyl Alcohol	Advanced refractory malignancies^d^	Dose escalation: 800, 1600 and 2400 mg/m^2^/dose administered 4 time a day	Gastrointestinal toxicities, including nausea, vomiting, satiety, and eructation, that were dose limiting	1200 mg/m^2^/dose	No anticancer activity observed in breast cancer patient. Tumor regression observed in one patient with metastatic colorectal cancer
Bailey et al. 2008 [25]	Phase 2: Women (*N* = 14)	Orally administered perillyl Alcohol	Advanced treatment-refractory breast cancer	Dose escalation: 1200–1500 mg/m^2^/dose administered 4 time a day	Poor toleration due to gastrointestinal and fatigue- related toxicities	Not indicated	No partial or complete regression observed in any participant.

^a^Including breast cancer colorectal carcinoma, metastatic adenocarcinoma, esophagus, pancreas, bronchus, ovary, and soft tissue sarcoma

^b^Partial response defined as ≥ 50% reduction in tumor size assessed by two measurements conducted ≥ 4 weeks apart

^c^Including prostrate (*n* = 4), ovarian (*n* = 3), sarcoma, renal cell (*n* = 3), breast (*n* = 2), hepatocellular (*n* = 2), chronic myelogenous leukemia (*n* = 1), chronic lymphocytic leukemia (*n* = 1),adenocarcinoma (*n* = 1)

^d^including: prostrate (*n* = 4), ovarian (*n* = 3), adenocarcinoma (*n* = 2), colorectal (*n* = 1), chronic myelogenous leukemia (*n* = 1), melanoma (*n* = 1), non-Hodgkin’s lymphoma (n-1), pancreas (*n* = 1), salivary gland (*n* = 1), and sarcoma (*n* = 1)

### Maximum tolerated dosing, toxicity and tolerance

Adverse events in all studies included in this review were classified according to the National Cancer Institute criteria and ranged from grade 0 (no events) to grade 4 (life threatening events). Overall, d-limonene was tolerable in both patients with advanced and early-stage malignancies, receiving single or multiple daily dosing [22, 26]. In the Vigushin et al. trial [22], 2 of the 32 participants receiving 6 g/m^2^/day discontinued the study as a result of limonene-linked gastrointestinal (GI) toxicity not exceeding grade 2 (nausea and diarrhea). A Maximum Tolerable Dose (MTD) of 8 g/m^2^/day was established in this study. However, dose escalation was limited by withdrawal from the studies due to disease progression. In the trial by Miller et al. [26], 3 of the 43 women terminated participation in the study early due to adverse events (heartburn, nausea and vomiting). In both the d-limonene trials, there were no grade 4 or serious organ toxicities observed.

Gastrointestinal toxicities were dose limiting in all three perillyl alcohol trials [23–25]. Dose-related adverse events including nausea, vomiting, diarrhea and fatigue were observed in these trials [23–25]. Two participants in the Ripple et al. (1998) trial [23] enrolled to level 3 dosing (2400 mg/m^2^/dose) required dose reduction to level 2 (1600 mg/m2/dose) due to these adverse events. Additionally, 3 participants experienced severe drug related myelosuppression – however, the three were ovarian (*n* = 2) and renal cell carcinoma (*n* = 1) cancer patients [23]. In the Ripple et al. (2000) trial [24], three participants experienced toxicities greater than grade 1 at 1200 and 1600 mg/m^2^/dose. This study established the MTD to be 1200 mg/m^2^/dose. This same MTD was arrived at by Bailey et al. trial [25] where 29 cycles of perillyl alcohol at 1200 mg/m^2^/dose were completed by all participants. Participants experienced majority of grade 3 and 4 toxicities in the first cycle. These included grade 3 nausea, vomiting, elevated alkaline phosphatase, and elevated aspartate transaminase; as well as grade 4 dyspnea and elevated lactate dehydrogenase [25]. Three participants whose dosing was escalated to 1500 mg/m^2^/dose, discontinued the study due to intolerability [25].

### Pharmacokinetic profile

Transformation of d-limonene into bioactive monoterpenes was observed in the trial by Vigushin et al. [22]. Metabolites, including perillic acid, dihydroperillic acid, limonene-1,2-diol, and uroterpenol, were observed, with peak concentration levels achieved on day 21 [22]. D-limonene was also found to accumulate in breast tissue (mean 41.3 μg/g tissue) and induce a statistically significant, albeit small, increase in IGF-I levels among study participants in the Miller et al. trial [26]. Furthermore, a statistically significant reduction (22%) in the tumor cyclin D1 expression was observed in this trial [26].

Perillyl alcohol metabolites including perillic acid dihydroperillic acid were detected in participant plasma, with peak levels occurring between 2 and 3 and 3–5 h after ingestion respectively [23]. Furthermore, about 9% of perillyl alcohol was excreted within the first 24 h through urine [23, 24].

### Anticancer activity

Overall, no objective complete tumor response – defined as absence of detectable clinical disease for more than 4 weeks – was observed in the d-limonene (*n* = 2) or perillyl alcohol (*n* = 3) trials. In the Vigushin et al. phase 1 d-limonene trial however, a partial response – defined as ≥ 50% reduction in tumor size assessed by two measurements conducted ≥ 4 weeks apart – was observed in one breast cancer patient [22]. This effect was sustained for 11 months and prompted a phase 2 trial exclusively among breast cancer patients. In this phase 2 trial (*n* = 10 participants), there was no response observed [22].

In the perillyl alcohol trials, no clinical benefit was observed among breast cancer patients [23–25]. In the Ripple et al. (2000) trial [24], however, chemotherapeutic activity was observed in 1 colorectal cancer patient, who experienced near-complete response – resolution of all but 1 lesion – for more than 2 years. Additionally, 2 prostate cancer patients treated at level 1 (800 mg/m^2^/dose) experienced disease stabilization for 13 and 10 months [24]. In the same study, a adenoidcystic carcinoma patient treated at level 2 (1200 mg/m^2^/dose) experienced stable disease for 8 months before progression [24]. Similarly, 2 metastatic breast patients in the Bailey et al. trial demonstrated disease stabilization [25]. However, there was no freedom from progression 1 year from initiation, with a median rate to disease progression of 35 days and a median survival of 389 days [25].

## Discussion

This scoping review aimed to explore the breadth and depth of existing evidence on the effect of d-limonene and its derivatives on breast cancer on human subjects, with the goal of highlighting gaps in knowledge. Our review yielded five eligible studies with a total of 133 participants, evaluating the chemotherapeutic properties of d-limonene (*n* = 2 trials; 85 participants) and perillyl alcohol (*n* = 3 trials; 48 participants). The number of articles resulting from our search was noticeably small, demonstrating the dearth of evidence available on the effect of d-limonene on breast cancer in human subjects. All studies included in this review were early-phase (1&2) clinical trials evaluating the safety and efficacy of the monoterpenes d-limonene and Perillyl alcohol. Perillyl alcohol dose escalation studies ranged from 800 to 2400 mg/m^2^/dose with a MTD of 1200 mg/m^2^/dose. This hydroxylated monoterpene derivative was generally poorly tolerated with participants experiencing dose-limiting gastrointestinal toxicities. Conversely, d-limonene was well tolerated in participants with early- and advance-stage malignancies who received doses ranging between 0.5 and 12 g/m^2^. Neither perillyl alcohol nor d-limonene demonstrated significant chemotherapeutic properties. We postulate that this null result may have been as a result of the study small sample sizes, and/or the advance stage malignancies of the participants.

In rodent models with chemically induced carcinogenesis, d-limonene and its derivatives have demonstrated a statistically significant decrease in mammary tumors incidence (Table 4) [16, 27, 28]. In one study, a 72% decrease in tumor incidence was observed among rats fed d-limonene (10,000 ppm) compared with controls not given the monoterpene and followed for 18 weeks [27]. Additionally, rodents fed d-limonene in the initiation phase showed an increase in the duration between introduction of the carcinogen and development of the first tumors [16, 29]. This increased latency period observed a dose response relationship, with longer latency at higher d-limonene dosage [27].

**Table 4:** Summary of findings from in vivo and in vitro pre-clinical studies evaluating the effect of d-limonene and its derivatives on breast cancer

Author(s)	Model	Compound and dosage under investigation	Cancer type	Effect
Elegbede et al. 1984 [27]	Female Sprague-Dawley Rats	1000 or 10,000 ppm d-limonene	DMBA^a^-induced rat mammary tumor	Inhibited mammary carcinogenesis due to increased latency; significant differences in incidence (72% reduction in tumors at 18 weeks among d-limonene fed animal); and regression of mammary tumors.
Elegbede et al.1986 [28]	Female (W/Fu X F344)F2	10% d-limonene	DMBA^a^-induced rat mammary tumor	Significant regression of chemically induced tumors in rats fed d-limonene (*p*-value 0.016). d-limonene inhibited formation of subsequent tumors (*p*-value < 0.025)
Elson et al. 1988 [29]	Female Sprague-Dawley Rats	5% d-limonene	DMBA^a^-induced rat mammary tumor	Reduction of average number of rat mammary carcinomas when fed d-limonene during the initiation or during the promotion/progression stage of carcinogenesis (*p* < 0.05); time to appearance of first tumor extended only when d-limonene fed during initiation stage (*p* < 0.005).
Maltzman et al 1989 [30].	Female Wistar-Furth rats	5% d-limonene and 5% orange oil	NMU^b^-induced mammary tumors	Orange oil (*p* < 0.001) and d-limonene (*p* < 0.001) prevent rat NMU- induced mammary carcinomas when introduced in the promotion/progression phase. No statistical difference in effect of orange oil and limonene.
Crowell et al. 1992 [16]	Female Wistar-Furth rats	1% d-limonene and 1% hydroxylated derivatives^c^	^a^DMBA-induced rat mammary tumor	No significant effect on tumor latency or multiplicity in rats receiving 1% d-limonene. Rats receiving 1% of uroterpenol and sobreol had significant increase in latency (*p* < 0.005 and *p* = 0.0001 respectively); significant decrease in tumor multiplicity in rats fed carveol (*p* < 0.05), uroterpenol (*p* < 0.025), and sobreol (*p* < 0.0001).
Haag et al. 1992 [31]	Female Wistar-Furth rats	0, 2.5, 5, 7.5 and 10% d-limonene	DMBA^a^ and NMU^b^ -induced rat mammary tumor	Statically significant complete regression rate observed starting at 5% limonene dietary levels. At 10% dietary limonene level, there was a 68% (*p* < 0.001) and 96% (*p* < 0.001) complete tumor regression rate in DBMA and NMU induced rats respectively. Established minimum dose of 7.5% dietary limonene required for a significant increase in complete tumor regression.
Jirtle et al. 1993 [32]	Female Fischer 344 rats	10% d-limonene	Advanced DMBA^a^-induced rat mammary tumor	Significant (*p* < 0.0001) regression in limonene fed rats (87%) compared to the control rodents (7%). Observed increased Growth Factor β1 and Mannose 6-Phosphate/Insulin-like Growth Factor II Receptor in limonene treated tumors, suggesting this as a possible mode of action.
Haag and Gould 1994 [15]	Female Wistar-Furth rats	2% Perillyl alcohol	DMBA^a^-induced rat mammary tumor	Statistically significant difference (*p* < 0.01) in complete regression of primary carcinomas (≥ 3 mm in diameter) of perillyl alcohol fed (2.5% w/w) rats (81% and the controls 31%). Treatment group also had lower rates of secondary tumors.
Chander et al. 1994 [33]	Female Ludwig/Wistar/Olac rats	10% limonene; 5% d-limonene; 12.5 mg/kg HAD^d^; and combination 5% d-limonene and 12.5 mg/kg HAD^d^	NMU^b^-induced mammary tumors	Significant rates of regression (*p* < 0.05) observed with 10% limonene; and 5% limonene, 4-HAD (12.5 mg/kg). Highest rate of tumor regression recorded in rats treated with the combination of 5% limonene and 4-HAD
Asamoto et al. 2002 [34]	Female Hras128^e^ rats	5% d-limonene	^b^NMU-induced mammary tumors	Significant reduction in multiplicity and tumor size (diameter *p* < 0.002, and volume *p* < 008) observed in rats treated with 5% d-limonene
Yuri et al. 2004 [35]	In vitro: ER^f^+ and ER^f^- human breast cancer cell lines In vivo: female BALB/c mice	500 *μ* M Perillyl alcohol for in vivo experiments; and 75 mg/Kg for in vitro studies	Human breast cancer cell inoculated mice	In vivo experiments: cell growth and proliferation inhibited by perillyl alcohol; In vitro study: treatment with perillyl alcohol resulted in significantly smaller tumors (*p* < 0.05) in terms of volume and weight

^a^DMBA – 7,12-Dimethylbenz[a]anthracene

^b^NMU- *N* -methyl- *N* -nitrosourea

^c^ Carveol, uroterpenol and sobrerol

^d^4-hydroxyandrostenedione

^e^Human c-Ha-ras proto-oncogene

^f^Estrogen receptor

When limonene derivatives were introduced in the promotion/progression stage in preclinical models, a statistically significant reduction in tumor multiplicity was observed [16]. Limonene, too, demonstrated a two-fold increase in protection against the development of secondary tumors, and induced a 63% regression in the secondary tumors formed [31]. Perillyl alcohol also demonstrated the prevention of development of secondary tumors [15] and suppressed growth in estrogen receptor human breast cancer cells [35]. Statistically significant reduction in tumor size (diameter *p* < 0.002, and volume *p* < 008) was observed in rats treated with 5% d-limonene [34].

Limonene-induced regression greater than 80% has been observed in 7,12-Dimethylbenz [a] anthracene (DBMA)-induced tumors, during the initiation and promotion/progression carcinogenesis stages [27–29, 31, 32]. Furthermore, studies examining perillyl alcohol demonstrated this limonene derivative induced regression in both early- and advance-stage carcinomas in DBMA-induced rodent models. In one study, a statistically significant difference (*p* < 0.01) in complete regression of primary carcinomas ≥ 3 mm in diameter of perillyl alcohol fed rats (81%) and the controls (31%) was observed [15]. However, in models where *N* -methyl- *N* -nitrosourea (NMU) was used as the carcinogen, tumor regression was only observed in the promotion/progression stage [30].

In DBMA and NMU cancer-induced rodent models, statistically significant levels of regression were observed beginning at 5% limonene dietary levels [31]. A dose of 7.5% was determined as the minimum dose required to observe significant increase in complete tumor regression in one study [31]. Given limited suppressive activity of limonene in the initiation phase of NMU-induced mammary carcinogenesis, Chander et al. assessed if supplementation with a aromatase inhibitor (4- hydroxyandrostenedione) could enhance tumor inhibition [33]. The researchers found that suboptimal doses of limonene and 4-HAD (5%) resulted in an 83% overall tumor regression (*p* < 0.001, 35]. Limited toxicity has been observed among rodent models treated with d-limonene and its derivatives [15, 31, 35].

Pre-clinical trials suggest serum levels of Transforming Growth Factor Beta 1 (TGF- *β*1) are linked to anticancer activity of monoterpenes through cell cycle regulation [32, 36]. Pharmacokinetic findings in this review demonstrated metabolic elements that are supported by these in vivo and in vitro findings. Miller et al. [26] reported a reduction in cyclin D1 expression, which has been shown to play a role in cell cycle progression [37–39]. The same study showed an increase in IGF-I among patients. While elevation of IGF-I is associated with increased cancer risk, the clinical implication in this study was unknown given the short duration of the study. A parallel study evaluating the plasma metabolic profiles of the same participants showed d-limonene as changing metabolic pathways in the participants [42]. Additionally, Pre-clinical studies have suggested that d-limonene induces cytostasis through selective inhibition of isopreylation of small G proteins [31].

None of the studies included in this review reported complete tumor response in patients with breast cancer. This null finding should be interpreted with caution for two reasons. First, most of the trials (4 of 5) were conducted among patients with advanced disease, who were heavily pretreated prior to enrolling in the respective studies [22–25]. Additionally, cancer subtype information was not reported in the studies included in this review. Preclinical findings may be an indication that perillyl alcohol may be chemopreventive, and not act during the progression stages of disease. Metabolic and pharmacokinetic analyses in the Miller et al. trial among newly diagnosed patients with operable breast cancer indicated more promising chemotherapeutic properties during this early-stage carcinogenesis [26]. Therefore, additional investigation of the effect of d-limonene and its derivatives in patients in the initiation stage of disease may be warranted. Furthermore, future studies should disaggregate findings by breast cancer subtype. Second, all studies recruited few patients, ranging from 10 to 43, which may have limited their power to detect clinically significant difference. Of note, the Bailey et al. [25] study was designed to enroll 40 participants to achieve adequate power. However, only 14 participants were enrolled [25]. Furthermore, the percentage of breast cancer patients enrolled in some studies was low. Notably, breast cancer participants constituted 11 and 6% of enrolled participants in the Ripple et al. (1998 and 2000) studies respectively [23, 24].

Of the two monoterpenes, d-limonene showed more chemotherapeutic promise, with partial tumor response observed in one patient [22]. Furthermore, d-limonene was found to be fat soluble, accumulating in breast tissue and retained in the body for a longer period of time [26]. This finding has been supported by other research, suggesting that d-limonene may be a candidate for further human trials for efficacy [10, 40]. Conversely, perillyl alcohol was expelled from the body at higher rates and did not show evidence of accumulation [23, 24]. This suggests that perillyl alcohol may need to be used in concert with other active ingredients, such as aromatase inhibitors, to bolster efficacy.

The present review was the first to systematically collate research on the effect of d-limonene and its derivatives on breast cancer in human subjects. However, our study was limited by the heterogeneity of the studies included in the review. Different indicators were used in the included trials, restricting our ability to compare chemotherapeutic activity across the studies.

## Conclusion

Citrus peel and citrus oils contain bioactive compounds, which laboratory and animal models have shown as promising to address breast cancer. This review of early clinical trials of d-limonene and its derivative, perillyl alcohol, demonstrated significant gaps in knowledge on the subject as evidenced by the few studies currently available. In the five trials included in the review, d-limonene (*n* = 2) was better tolerated and exhibited more promising chemopreventive properties compared to its derivative (*n* = 3). Well-powered clinical trials of d-limonene among patients with early-stage carcinogenesis may offer greater insight into the effect of d-limonene on breast cancer.

## Supplementary Information

**Additional file 1: Table S1**. Databases and search terms used for scoping review

**Additional file 2: Table S2.** JBI Critical appraisal checklist for non-randomized experimental studies*.

**Additional file 3: Table S3.** PRISMA-ScR checklist*.

## Data Availability

Not applicable.

## Abbreviations

ACA: American Cancer Association
CINAHL: Cumulative Index of Nursing and Allied Health Literature
DMBA: 7,12-Dimethylbenz [a]anthracene
GERD: Gastroesophageal Reflux Disease
GI: Gastrointestinal
JBI: Joanna Briggs Institute
MTD: Maximum Tolerable Dose
NMU: *N* -methyl- *N* -nitrosourea
PRISMA-ScR: PRISMA Extension for Scoping Reviews
ROBINS-I: Risk Of Bias In Non-randomized Studies of Interventions
TGF- *β*1: Transforming Growth Factor Beta 1
