# Supplementary material for: Effect of d-limonene and its derivatives on breast cancer in human trials: a scoping review and narrative synthesis
Source: BMC Cancer. 2021 Aug 6;21:902. doi: 10.1186/s12885-021-08639-1 (PMC8349000; doi:10.1186/s12885-021-08639-1)
Supplement: Supplementary file 1 — Additional file 1: Table S1. Databases and search terms used for scoping review [file 12885_2021_8639_MOESM1_ESM.docx]

**Supplemental table 1**: Databases and search terms used for scoping review

| **Database** | **Keywords** |
| --- | --- |
| PubMed | (Breast cancer OR breast carcinoma OR mammary cancer OR cancer of the breast OR “breast neoplasms”[mesh])  AND  (d-limonene OR citrus oil OR orange oil OR Lemon oil OR Mandarin oil OR Lime oil OR Grapefruit oil OR citrus peel OR carveol OR uroterpenol OR sobrerol OR  “limonene”[mesh] OR “citrus”[mesh] OR “citrus paradise”[mesh]) |
| Embase | (‘breast cancer’ OR ‘breast carcinoma’ OR ‘mammary cancer’ OR ‘cancer of the breast’ OR ‘breast cancer’/exp)  AND  ('d limonene' OR 'citrus oil' OR 'orange oil' OR 'lemon oil' OR 'mandarin oil' OR 'lime oil' OR 'grapefruit oil' OR 'd limonene'/exp OR 'citrus peel' OR 'lemon oil'/exp OR 'citrus oil'/exp OR carveol OR uroterpenol OR sobrerol OR ‘carveol’/exp OR ‘sobrerol’/exp) |
| CINAHL | (“Breast cancer” OR “breast carcinoma” OR “mammary cancer” OR “cancer of the breast” OR (MH "Breast Neoplasms"))  AND  (“d-limonene” OR “citrus oil” OR “orange oil” OR “lemon oil” OR “mandarin oil” OR “lime oil” OR “grapefruit oil” OR carveol OR uroterpenol OR sobrerol) |
| WoS Core | TS=(“breast cancer” OR “breast carcinoma” OR “mammary cancer” OR “cancer of the breast”)  AND  TS=(“d-limonene” OR “citrus oil” OR “orange oil” OR “lemon oil” OR “mandarin oil” OR “lime oil” OR “grapefruit oil” OR carveol OR uroterpenol OR sobrerol) |
| Cochrane Reviews | (Breast cancer OR breast carcinoma OR mammary cancer OR cancer of the breast)  AND  (d-limonene OR citrus oil OR orange oil OR Lemon oil OR Mandarin oil OR Lime oil OR Grapefruit oil OR citrus peel OR carveol OR uroterpenol OR sobrerol) |
