# Supplementary material for: Effect of d-limonene and its derivatives on breast cancer in human trials: a scoping review and narrative synthesis
Source: BMC Cancer. 2021 Aug 6;21:902. doi: 10.1186/s12885-021-08639-1 (PMC8349000; doi:10.1186/s12885-021-08639-1)
Supplement: Supplementary file 2 — Additional file 2: Table S2. JBI Critical appraisal checklist for non-randomized experimental studies*. [file 12885_2021_8639_MOESM2_ESM.docx]

**Supplemental table 1:** JBI Critical appraisal checklist for non-randomized experimental studies*****

| **Criteria** | Vigushin *et al.* 1998 [22] | Ripple *et al.* 1998 [25] | Ripple *et al.* 2000 [26] | Bailey *et al.* 2008 [23] | Miller *et al.* 2013 [24] |
| --- | --- | --- | --- | --- | --- |
| Is it clear in the study what is the ‘cause’ and what is the ‘effect’ (i.e. there is no confusion about which variable comes first) | ✓ | ✓ | ✓ | ✓ | ✓ |
| Were the participants included in any comparisons similar? | ✓ | ✓ | ✓ | ✓ | ✓ |
| Were the participants included in any comparisons receiving similar treatment/care, other than the exposure or intervention of interest? | ✓ | ✓ | ✓ | ✓ | ✓ |
| Was there a control group? | 🗶 | 🗶 | 🗶 | 🗶 | 🗶 |
| Were there multiple measurements of the outcome both pre and post the intervention/exposure? | ✓ | ✓ | ✓ | ✓ | ✓ |
| Was follow up complete and if not, were differences between groups in terms of their follow up adequately described and analyzed? | ✓ | ✓ | ✓ | ✓ | ✓ |
| Were the outcomes of participants included in any comparisons measured in the same way? | ✓ | ✓ | ✓ | ✓ | ✓ |
| Were outcomes measured in a reliable way? | ✓ | ✓ | ✓ | ✓ | ✓ |
| Was appropriate statistical analysis used? | ✓ | ✓ | ✓ | ✓ | ✓ |
| **Overall appraisal** | Include | Include | Include | Include | Include |

* Tufanaru C, Munn Z, Aromataris E, Campbell J, Hopp L. Chapter 3: Systematic reviews of effectiveness. In: Aromataris E, Munn Z (Editors). Joanna Briggs Institute Reviewer's Manual.The Joanna Briggs Institute, 2017. Available fromhttps://reviewersmanual.joannabriggs.org/
